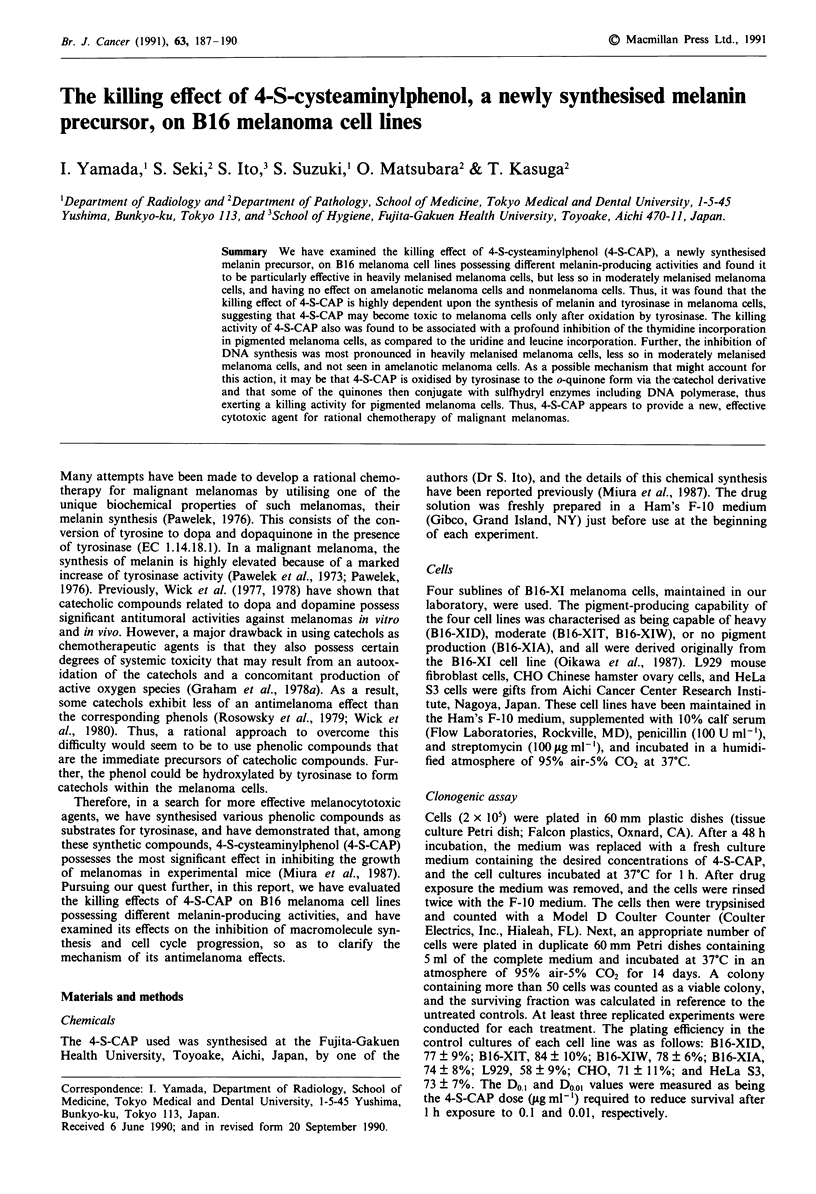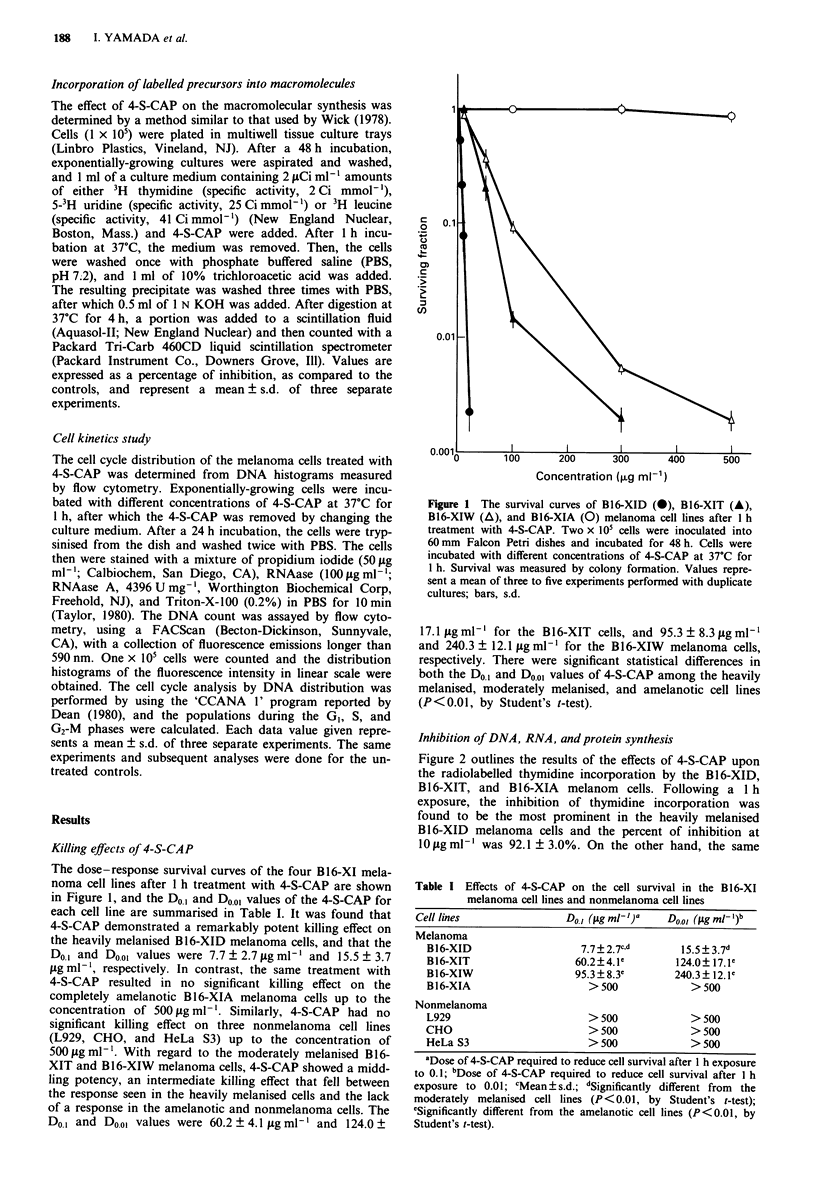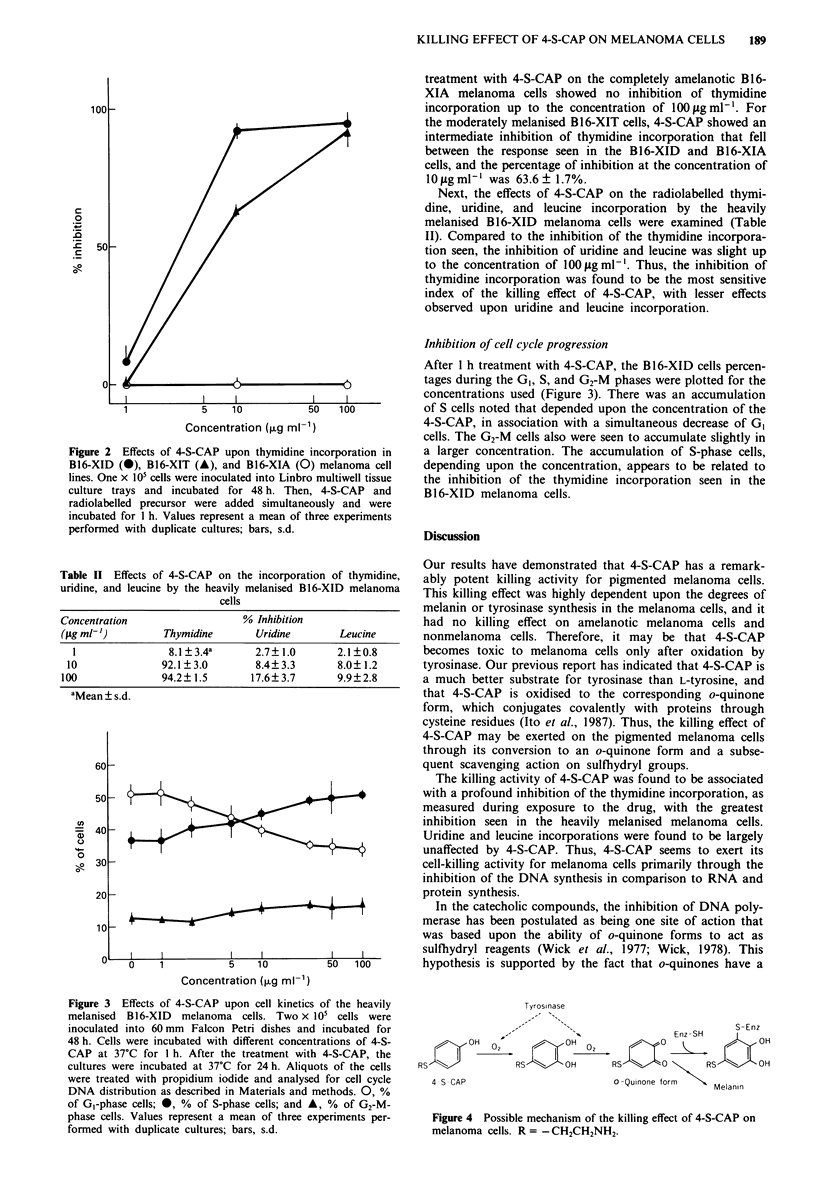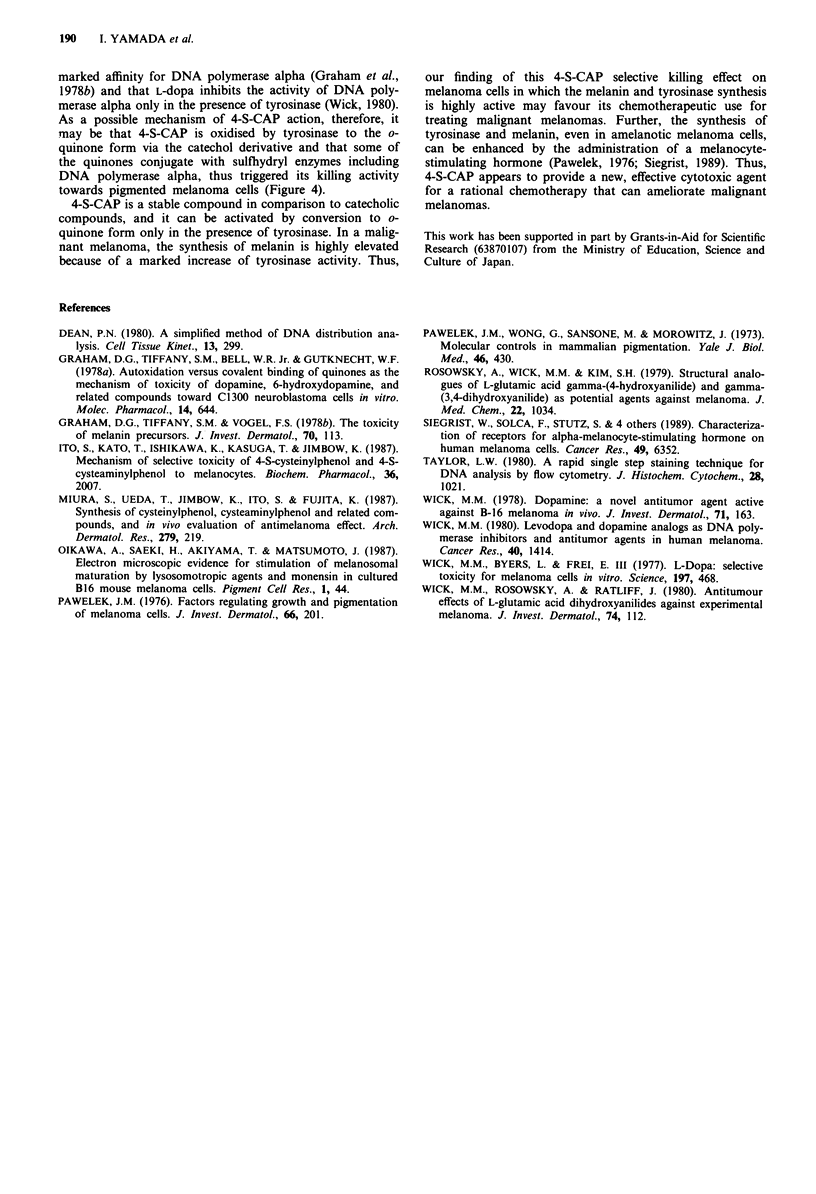# The killing effect of 4-S-cysteaminylphenol, a newly synthesised melanin precursor, on B16 melanoma cell lines.

**DOI:** 10.1038/bjc.1991.46

**Published:** 1991-02

**Authors:** I. Yamada, S. Seki, S. Ito, S. Suzuki, O. Matsubara, T. Kasuga

**Affiliations:** Department of Radiology, School of Medicine, Tokyo Medical and Dental University, Japan.

## Abstract

We have examined the killing effect of 4-S-cysteaminylphenol (4-S-CAP), a newly synthesised melanin precursor, on B16 melanoma cell lines possessing different melanin-producing activities and found it to be particularly effective in heavily melanised melanoma cells, but less so in moderately melanised melanoma cells, and having no effect on amelanotic melanoma cells and nonmelanoma cells. Thus, it was found that the killing effect of 4-S-CAP is highly dependent upon the synthesis of melanin and tyrosinase in melanoma cells, suggesting that 4-S-CAP may become toxic to melanoma cells only after oxidation by tyrosinase. The killing activity of 4-S-CAP also was found to be associated with a profound inhibition of the thymidine incorporation in pigmented melanoma cells, as compared to the uridine and leucine incorporation. Further, the inhibition of DNA synthesis was most pronounced in heavily melanised melanoma cells, less so in moderately melanised melanoma cells, and not seen in amelanotic melanoma cells. As a possible mechanism that might account for this action, it may be that 4-S-CAP is oxidised by tyrosinase to the o-quinone form via the catechol derivative and that some of the quinones then conjugate with sulfhydryl enzymes including DNA polymerase, thus exerting a killing activity for pigmented melanoma cells. Thus, 4-S-CAP appears to provide a new, effective cytotoxic agent for rational chemotherapy of malignant melanomas.


*Br. J. Cancer* (1991), **63**, 187–190 © Macmillan Press Ltd., 1991

### The killing effect of 4-S-cysteaminylphenol, a newly synthesised melanin precursor, on B16 melanoma cell lines

I. Yamada,[1] S. Seki,[2] S. Ito,[3] S. Suzuki,[1] O. Matsubara[2] & T. Kasuga[2]

[1]*Department of Radiology and* [2]*Department of Pathology, School of Medicine, Tokyo Medical and Dental University, 1-5-45 Yushima, Bunkyo-ku, Tokyo 113, and* [3]*School of Hygiene, Fujita-Gakuen Health University, Toyoake, Aichi 470-11, Japan.*

**Summary** We have examined the killing effect of 4-S-cysteaminylphenol (4-S-CAP), a newly synthesised melanin precursor, on B16 melanoma cell lines possessing different melanin-producing activities and found it to be particularly effective in heavily melanised melanoma cells, but less so in moderately melanised melanoma cells, and having no effect on amelanotic melanoma cells and nonmelanoma cells. Thus, it was found that the killing effect of 4-S-CAP is highly dependent upon the synthesis of melanin and tyrosinase in melanoma cells, suggesting that 4-S-CAP may become toxic to melanoma cells only after oxidation by tyrosinase. The killing activity of 4-S-CAP also was found to be associated with a profound inhibition of the thymidine incorporation in pigmented melanoma cells, as compared to the uridine and leucine incorporation. Further, the inhibition of DNA synthesis was most pronounced in heavily melanised melanoma cells, less so in moderately melanised melanoma cells, and not seen in amelanotic melanoma cells. As a possible mechanism that might account for this action, it may be that 4-S-CAP is oxidised by tyrosinase to the *o*-quinone form via the catechol derivative and that some of the quinones then conjugate with sulfhydryl enzymes including DNA polymerase, thus exerting a killing activity for pigmented melanoma cells. Thus, 4-S-CAP appears to provide a new, effective cytotoxic agent for rational chemotherapy of malignant melanomas.

Many attempts have been made to develop a rational chemotherapy for malignant melanomas by utilising one of the unique biochemical properties of such melanomas, their melanin synthesis (Pawelek, 1976). This consists of the conversion of tyrosine to dopa and dopaquinone in the presence of tyrosinase (EC 1.14.18.1). In a malignant melanoma, the synthesis of melanin is highly elevated because of a marked increase of tyrosinase activity (Pawelek *et al.*, 1973; Pawelek, 1976). Previously, Wick *et al.* (1977, 1978) have shown that catecholic compounds related to dopa and dopamine possess significant antitumoral activities against melanomas *in vitro* and *in vivo*. However, a major drawback in using catechols as chemotherapeutic agents is that they also possess certain degrees of systemic toxicity that may result from an autooxidation of the catechols and a concomitant production of active oxygen species (Graham *et al.*, 1978*a*). As a result, some catechols exhibit less of an antimelanoma effect than the corresponding phenols (Rosowsky *et al.*, 1979; Wick *et al.*, 1980). Thus, a rational approach to overcome this difficulty would seem to be to use phenolic compounds that are the immediate precursors of catecholic compounds. Further, the phenol could be hydroxylated by tyrosinase to form catechols within the melanoma cells.

Therefore, in a search for more effective melanocytotoxic agents, we have synthesised various phenolic compounds as substrates for tyrosinase, and have demonstrated that, among these synthetic compounds, 4-S-cysteaminylphenol (4-S-CAP) possesses the most significant effect in inhibiting the growth of melanomas in experimental mice (Miura *et al.*, 1987). Pursuing our quest further, in this report, we have evaluated the killing effects of 4-S-CAP on B16 melanoma cell lines possessing different melanin-producing activities, and have examined its effects on the inhibition of macromolecule synthesis and cell cycle progression, so as to clarify the mechanism of its antimelanoma effects.

### Materials and methods

### *Chemicals*

The 4-S-CAP used was synthesised at the Fujita-Gakuen Health University, Toyoake, Aichi, Japan, by one of the authors (Dr S. Ito), and the details of this chemical synthesis have been reported previously (Miura *et al.*, 1987). The drug solution was freshly prepared in a Ham's F-10 medium (Gibco, Grand Island, NY) just before use at the beginning of each experiment.

### *Cells*

Four sublines of B16-XI melanoma cells, maintained in our laboratory, were used. The pigment-producing capability of the four cell lines was characterised as being capable of heavy (B16-XID), moderate (B16-XIT, B16-XIW), or no pigment production (B16-XIA), and all were derived originally from the B16-XI cell line (Oikawa *et al.*, 1987). L929 mouse fibroblast cells, CHO Chinese hamster ovary cells, and HeLa S3 cells were gifts from Aichi Cancer Center Research Institute, Nagoya, Japan. These cell lines have been maintained in the Ham's F-10 medium, supplemented with 10% calf serum (Flow Laboratories, Rockville, MD), penicillin (100 U $ml^{-1}$), and streptomycin (100 $\mu g\ ml^{-1}$), and incubated in a humidified atmosphere of 95% air-5% $CO_2$ at 37°C.

### *Clonogenic assay*

Cells ($2 \times 10^5$) were plated in 60 mm plastic dishes (tissue culture Petri dish; Falcon plastics, Oxnard, CA). After a 48 h incubation, the medium was replaced with a fresh culture medium containing the desired concentrations of 4-S-CAP, and the cell cultures incubated at 37°C for 1 h. After drug exposure the medium was removed, and the cells were rinsed twice with the F-10 medium. The cells then were trypsinised and counted with a Model D Coulter Counter (Coulter Electrics, Inc., Hialeah, FL). Next, an appropriate number of cells were plated in duplicate 60 mm Petri dishes containing 5 ml of the complete medium and incubated at 37°C in an atmosphere of 95% air-5% $CO_2$ for 14 days. A colony containing more than 50 cells was counted as a viable colony, and the surviving fraction was calculated in reference to the untreated controls. At least three replicated experiments were conducted for each treatment. The plating efficiency in the control cultures of each cell line was as follows: B16-XID, 77 ± 9%; B16-XIT, 84 ± 10%; B16-XIW, 78 ± 6%; B16-XIA, 74 ± 8%; L929, 58 ± 9%; CHO, 71 ± 11%; and HeLa S3, 73 ± 7%. The $D_{0.1}$ and $D_{0.01}$ values were measured as being the 4-S-CAP dose ($\mu g\ ml^{-1}$) required to reduce survival after 1 h exposure to 0.1 and 0.01, respectively.

Correspondence: I. Yamada, Department of Radiology, School of Medicine, Tokyo Medical and Dental University, 1-5-45 Yushima, Bunkyo-ku, Tokyo 113, Japan.
Received 6 June 1990; and in revised form 20 September 1990.

*Incorporation of labelled precursors into macromolecules*

The effect of 4-S-CAP on the macromolecular synthesis was determined by a method similar to that used by Wick (1978). Cells ($1 \times 10^5$) were plated in multiwell tissue culture trays (Linbro Plastics, Vineland, NJ). After a 48 h incubation, exponentially-growing cultures were aspirated and washed, and 1 ml of a culture medium containing 2 $\mu$Ci ml$^{-1}$ amounts of either $^3$H thymidine (specific activity, 2 Ci mmol$^{-1}$), 5-$^3$H uridine (specific activity, 25 Ci mmol$^{-1}$) or $^3$H leucine (specific activity, 41 Ci mmol$^{-1}$) (New England Nuclear, Boston, Mass.) and 4-S-CAP were added. After 1 h incubation at 37°C, the medium was removed. Then, the cells were washed once with phosphate buffered saline (PBS, pH 7.2), and 1 ml of 10% trichloroacetic acid was added. The resulting precipitate was washed three times with PBS, after which 0.5 ml of 1 N KOH was added. After digestion at 37°C for 4 h, a portion was added to a scintillation fluid (Aquasol-II; New England Nuclear) and then counted with a Packard Tri-Carb 460CD liquid scintillation spectrometer (Packard Instrument Co., Downers Grove, Ill). Values are expressed as a percentage of inhibition, as compared to the controls, and represent a mean ± s.d. of three separate experiments.

*Cell kinetics study*

The cell cycle distribution of the melanoma cells treated with 4-S-CAP was determined from DNA histograms measured by flow cytometry. Exponentially-growing cells were incubated with different concentrations of 4-S-CAP at 37°C for 1 h, after which the 4-S-CAP was removed by changing the culture medium. After a 24 h incubation, the cells were trypsinised from the dish and washed twice with PBS. The cells then were stained with a mixture of propidium iodide (50 $\mu$g ml$^{-1}$; Calbiochem, San Diego, CA), RNAase (100 $\mu$g ml$^{-1}$; RNAase A, 4396 U mg$^{-1}$, Worthington Biochemical Corp, Freehold, NJ), and Triton-X-100 (0.2%) in PBS for 10 min (Taylor, 1980). The DNA count was assayed by flow cytometry, using a FACScan (Becton-Dickinson, Sunnyvale, CA), with a collection of fluorescence emissions longer than 590 nm. One $\times 10^5$ cells were counted and the distribution histograms of the fluorescence intensity in linear scale were obtained. The cell cycle analysis by DNA distribution was performed by using the 'CCANA 1' program reported by Dean (1980), and the populations during the $G_1$, S, and $G_2$-M phases were calculated. Each data value given represents a mean ± s.d. of three separate experiments. The same experiments and subsequent analyses were done for the untreated controls.

### Results

*Killing effects of 4-S-CAP*

The dose–response survival curves of the four B16-XI melanoma cell lines after 1 h treatment with 4-S-CAP are shown in Figure 1, and the $D_{0.1}$ and $D_{0.01}$ values of the 4-S-CAP for each cell line are summarised in Table I. It was found that 4-S-CAP demonstrated a remarkably potent killing effect on the heavily melanised B16-XID melanoma cells, and that the $D_{0.1}$ and $D_{0.01}$ values were 7.7 ± 2.7 $\mu$g ml$^{-1}$ and 15.5 ± 3.7 $\mu$g ml$^{-1}$, respectively. In contrast, the same treatment with 4-S-CAP resulted in no significant killing effect on the completely amelanotic B16-XIA melanoma cells up to the concentration of 500 $\mu$g ml$^{-1}$. Similarly, 4-S-CAP had no significant killing effect on three nonmelanoma cell lines (L929, CHO, and HeLa S3) up to the concentration of 500 $\mu$g ml$^{-1}$. With regard to the moderately melanised B16-XIT and B16-XIW melanoma cells, 4-S-CAP showed a middling potency, an intermediate killing effect that fell between the response seen in the heavily melanised cells and the lack of a response in the amelanotic and nonmelanoma cells. The $D_{0.1}$ and $D_{0.01}$ values were 60.2 ± 4.1 $\mu$g ml$^{-1}$ and 124.0 ± 17.1 $\mu$g ml$^{-1}$ for the B16-XIT cells, and 95.3 ± 8.3 $\mu$g ml$^{-1}$ and 240.3 ± 12.1 $\mu$g ml$^{-1}$ for the B16-XIW melanoma cells, respectively. There were significant statistical differences in both the $D_{0.1}$ and $D_{0.01}$ values of 4-S-CAP among the heavily melanised, moderately melanised, and amelanotic cell lines ($P < 0.01$, by Student's $t$-test).

**Figure 1** The survival curves of B16-XID (●), B16-XIT (▲), B16-XIW (Δ), and B16-XIA (O) melanoma cell lines after 1 h treatment with 4-S-CAP. Two $\times 10^5$ cells were inoculated into 60 mm Falcon Petri dishes and incubated for 48 h. Cells were incubated with different concentrations of 4-S-CAP at 37°C for 1 h. Survival was measured by colony formation. Values represent a mean of three to five experiments performed with duplicate cultures; bars, s.d.

*Inhibition of DNA, RNA, and protein synthesis*

Figure 2 outlines the results of the effects of 4-S-CAP upon the radiolabelled thymidine incorporation by the B16-XID, B16-XIT, and B16-XIA melanom cells. Following a 1 h exposure, the inhibition of thymidine incorporation was found to be the most prominent in the heavily melanised B16-XID melanoma cells and the percent of inhibition at 10 $\mu$g ml$^{-1}$ was 92.1 ± 3.0%. On the other hand, the same

**Table I** Effects of 4-S-CAP on the cell survival in the B16-XI melanoma cell lines and nonmelanoma cell lines

| *Cell lines* | $D_{0.1}$ *(μg ml$^{-1}$)*[a] | $D_{0.01}$ *(μg ml$^{-1}$)*[b] |
|---|---|---|
| Melanoma | | |
| B16-XID | 7.7±2.7[c,d] | 15.5±3.7[d] |
| B16-XIT | 60.2±4.1[e] | 124.0±17.1[e] |
| B16-XIW | 95.3±8.3[e] | 240.3±12.1[e] |
| B16-XIA | >500 | >500 |
| Nonmelanoma | | |
| L929 | >500 | >500 |
| CHO | >500 | >500 |
| HeLa S3 | >500 | >500 |

[a]Dose of 4-S-CAP required to reduce cell survival after 1 h exposure to 0.1; [b]Dose of 4-S-CAP required to reduce cell survival after 1 h exposure to 0.01; [c]Mean±s.d.; [d]Significantly different from the moderately melanised cell lines ($P < 0.01$, by Student's $t$-test); [e]Significantly different from the amelanotic cell lines ($P < 0.01$, by Student's $t$-test).

**Figure 2** Effects of 4-S-CAP upon thymidine incorporation in B16-XID (●), B16-XIT (▲), and B16-XIA (O) melanoma cell lines. One × $10^5$ cells were inoculated into Linbro multiwell tissue culture trays and incubated for 48 h. Then, 4-S-CAP and radiolabelled precursor were added simultaneously and were incubated for 1 h. Values represent a mean of three experiments performed with duplicate cultures; bars, s.d.

**Table II** Effects of 4-S-CAP on the incorporation of thymidine, uridine, and leucine by the heavily melanised B16-XID melanoma cells

| *Concentration* | | *% Inhibition* | |
|---|---|---|---|
| *(μg ml⁻¹)* | *Thymidine* | *Uridine* | *Leucine* |
| 1 | 8.1±3.4[a] | 2.7±1.0 | 2.1±0.8 |
| 10 | 92.1±3.0 | 8.4±3.3 | 8.0±1.2 |
| 100 | 94.2±1.5 | 17.6±3.7 | 9.9±2.8 |

[a]Mean±s.d.

**Figure 3** Effects of 4-S-CAP upon cell kinetics of the heavily melanised B16-XID melanoma cells. Two × $10^5$ cells were inoculated into 60 mm Falcon Petri dishes and incubated for 48 h. Cells were incubated with different concentrations of 4-S-CAP at 37°C for 1 h. After the treatment with 4-S-CAP, the cultures were incubated at 37°C for 24 h. Aliquots of the cells were treated with propidium iodide and analysed for cell cycle DNA distribution as described in Materials and methods. O, % of $G_1$-phase cells; ●, % of S-phase cells; and ▲, % of $G_2$-M-phase cells. Values represent a mean of three experiments performed with duplicate cultures; bars, s.d.

treatment with 4-S-CAP on the completely amelanotic B16-XIA melanoma cells showed no inhibition of thymidine incorporation up to the concentration of $100\,\mu g\,ml^{-1}$. For the moderately melanised B16-XIT cells, 4-S-CAP showed an intermediate inhibition of thymidine incorporation that fell between the response seen in the B16-XID and B16-XIA cells, and the percentage of inhibition at the concentration of $10\,\mu g\,ml^{-1}$ was $63.6 \pm 1.7\%$.

Next, the effects of 4-S-CAP on the radiolabelled thymidine, uridine, and leucine incorporation by the heavily melanised B16-XID melanoma cells were examined (Table II). Compared to the inhibition of the thymidine incorporation seen, the inhibition of uridine and leucine was slight up to the concentration of $100\,\mu g\,ml^{-1}$. Thus, the inhibition of thymidine incorporation was found to be the most sensitive index of the killing effect of 4-S-CAP, with lesser effects observed upon uridine and leucine incorporation.

*Inhibition of cell cycle progression*

After 1 h treatment with 4-S-CAP, the B16-XID cells percentages during the $G_1$, S, and $G_2$-M phases were plotted for the concentrations used (Figure 3). There was an accumulation of S cells noted that depended upon the concentration of the 4-S-CAP, in association with a simultaneous decrease of $G_1$ cells. The $G_2$-M cells also were seen to accumulate slightly in a larger concentration. The accumulation of S-phase cells, depending upon the concentration, appears to be related to the inhibition of the thymidine incorporation seen in the B16-XID melanoma cells.

### Discussion

Our results have demonstrated that 4-S-CAP has a remarkably potent killing activity for pigmented melanoma cells. This killing effect was highly dependent upon the degrees of melanin or tyrosinase synthesis in the melanoma cells, and it had no killing effect on amelanotic melanoma cells and nonmelanoma cells. Therefore, it may be that 4-S-CAP becomes toxic to melanoma cells only after oxidation by tyrosinase. Our previous report has indicated that 4-S-CAP is a much better substrate for tyrosinase than L-tyrosine, and that 4-S-CAP is oxidised to the corresponding *o*-quinone form, which conjugates covalently with proteins through cysteine residues (Ito *et al.*, 1987). Thus, the killing effect of 4-S-CAP may be exerted on the pigmented melanoma cells through its conversion to an *o*-quinone form and a subsequent scavenging action on sulfhydryl groups.

The killing activity of 4-S-CAP was found to be associated with a profound inhibition of the thymidine incorporation, as measured during exposure to the drug, with the greatest inhibition seen in the heavily melanised melanoma cells. Uridine and leucine incorporations were found to be largely unaffected by 4-S-CAP. Thus, 4-S-CAP seems to exert its cell-killing activity for melanoma cells primarily through the inhibition of the DNA synthesis in comparison to RNA and protein synthesis.

In the catecholic compounds, the inhibition of DNA polymerase has been postulated as being one site of action that was based upon the ability of *o*-quinone forms to act as sulfhydryl reagents (Wick *et al.*, 1977; Wick, 1978). This hypothesis is supported by the fact that *o*-quinones have a

**Figure 4** Possible mechanism of the killing effect of 4-S-CAP on melanoma cells. R = $-CH_2CH_2NH_2$.

marked affinity for DNA polymerase alpha (Graham *et al.*, 1978*b*) and that L-dopa inhibits the activity of DNA polymerase alpha only in the presence of tyrosinase (Wick, 1980). As a possible mechanism of 4-S-CAP action, therefore, it may be that 4-S-CAP is oxidised by tyrosinase to the *o*-quinone form via the catechol derivative and that some of the quinones conjugate with sulfhydryl enzymes including DNA polymerase alpha, thus triggered its killing activity towards pigmented melanoma cells (Figure 4).

4-S-CAP is a stable compound in comparison to catecholic compounds, and it can be activated by conversion to *o*-quinone form only in the presence of tyrosinase. In a malignant melanoma, the synthesis of melanin is highly elevated because of a marked increase of tyrosinase activity. Thus, our finding of this 4-S-CAP selective killing effect on melanoma cells in which the melanin and tyrosinase synthesis is highly active may favour its chemotherapeutic use for treating malignant melanomas. Further, the synthesis of tyrosinase and melanin, even in amelanotic melanoma cells, can be enhanced by the administration of a melanocyte-stimulating hormone (Pawelek, 1976; Siegrist, 1989). Thus, 4-S-CAP appears to provide a new, effective cytotoxic agent for a rational chemotherapy that can ameliorate malignant melanomas.

This work has been supported in part by Grants-in-Aid for Scientific Research (63870107) from the Ministry of Education, Science and Culture of Japan.

### References

DEAN, P.N. (1980). A simplified method of DNA distribution analysis. *Cell Tissue Kinet.*, **13**, 299.

GRAHAM, D.G., TIFFANY, S.M., BELL, W.R. Jr. & GUTKNECHT, W.F. (1978*a*). Autoxidation versus covalent binding of quinones as the mechanism of toxicity of dopamine, 6-hydroxydopamine, and related compounds toward C1300 neuroblastoma cells *in vitro*. *Molec. Pharmacol.*, **14**, 644.

GRAHAM, D.G., TIFFANY, S.M. & VOGEL, F.S. (1978*b*). The toxicity of melanin precursors. *J. Invest. Dermatol.*, **70**, 113.

ITO, S., KATO, T., ISHIKAWA, K., KASUGA, T. & JIMBOW, K. (1987). Mechanism of selective toxicity of 4-S-cysteinylphenol and 4-S-cysteaminylphenol to melanocytes. *Biochem. Pharmacol.*, **36**, 2007.

MIURA, S., UEDA, T., JIMBOW, K., ITO, S. & FUJITA, K. (1987). Synthesis of cysteinylphenol, cysteaminylphenol and related compounds, and *in vivo* evaluation of antimelanoma effect. *Arch. Dermatol. Res.*, **279**, 219.

OIKAWA, A., SAEKI, H., AKIYAMA, T. & MATSUMOTO, J. (1987). Electron microscopic evidence for stimulation of melanosomal maturation by lysosomotropic agents and monensin in cultured B16 mouse melanoma cells. *Pigment Cell Res.*, **1**, 44.

PAWELEK, J.M. (1976). Factors regulating growth and pigmentation of melanoma cells. *J. Invest. Dermatol.*, **66**, 201.

PAWELEK, J.M., WONG, G., SANSONE, M. & MOROWITZ, J. (1973). Molecular controls in mammalian pigmentation. *Yale J. Biol. Med.*, **46**, 430.

ROSOWSKY, A., WICK, M.M. & KIM, S.H. (1979). Structural analogues of L-glutamic acid gamma-(4-hydroxyanilide) and gamma-(3,4-dihydroxyanilide) as potential agents against melanoma. *J. Med. Chem.*, **22**, 1034.

SIEGRIST, W., SOLCA, F., STUTZ, S. & 4 others (1989). Characterization of receptors for alpha-melanocyte-stimulating hormone on human melanoma cells. *Cancer Res.*, **49**, 6352.

TAYLOR, L.W. (1980). A rapid single step staining technique for DNA analysis by flow cytometry. *J. Histochem. Cytochem.*, **28**, 1021.

WICK, M.M. (1978). Dopamine: a novel antitumor agent active against B-16 melanoma *in vivo*. *J. Invest. Dermatol.*, **71**, 163.

WICK, M.M. (1980). Levodopa and dopamine analogs as DNA polymerase inhibitors and antitumor agents in human melanoma. *Cancer Res.*, **40**, 1414.

WICK, M.M., BYERS, L. & FREI, E. III (1977). L-Dopa: selective toxicity for melanoma cells *in vitro*. *Science*, **197**, 468.

WICK, M.M., ROSOWSKY, A. & RATLIFF, J. (1980). Antitumour effects of L-glutamic acid dihydroxyanilides against experimental melanoma. *J. Invest. Dermatol.*, **74**, 112.